# Dietary Inflammatory Potential and Sociodemographic Correlates Among Adults in Saudi Arabia: A Cross-Sectional Study

**DOI:** 10.3390/nu17243851

**Published:** 2025-12-10

**Authors:** Raneem Asiri, Shoug Alashmali

**Affiliations:** Department of Clinical Nutrition, Faculty of Applied Medical Sciences, King Abdulaziz University, Jeddah 21589, Saudi Arabia; rasiri0115@stu.kau.edu.sa

**Keywords:** diet, inflammation, sociodemographic, adults, Saudi Arabia

## Abstract

**Background**: Dietary patterns influence systemic inflammation, which is involved in the pathogenesis of non-communicable diseases. The dietary inflammatory index (DII) quantifies the inflammatory potential of the diet and varies across populations due to cultural and regional eating habits. Limited data exist on the inflammatory potential of diets in Saudi Arabia. This study aimed to assess the inflammatory potential of the diet and its association with sociodemographic and lifestyle factors among adults in Saudi Arabia. **Methods**: A cross-sectional study included 256 adults aged 18–50 years residing in Saudi Arabia. Participants were recruited using convenience sampling via social media platforms. Data were collected between November 2024 and August 2025 using a validated Saudi food frequency questionnaire and sociodemographic survey. Energy-adjusted DII (E-DII) scores were calculated using 42 food parameters. Non-parametric tests (Mann–Whitney U and Kruskal–Wallis) were applied to examine associations between E-DII and sociodemographic variables. **Results**: Significant differences in E-DII were observed by sex (*p* < 0.001). Males had higher E-DII scores than females, suggesting more pro-inflammatory diets. Participants with postgraduate education tended to have lower E-DII than participants with only a high school degree, reflecting more anti-inflammatory dietary patterns. However, this trend was not statistically significant (*p* = 0.06). The mean E-DII was 4.8 ± 1.3, indicating a predominantly pro-inflammatory dietary pattern. No significant differences were found across age, education, income, BMI, marital status, employment, or smoking status. **Conclusions**: Sex was a key determinant of dietary inflammatory potential. Adults demonstrated overall pro-inflammatory dietary patterns in Saudi Arabia. Public health interventions should target higher risk groups, such as males with a higher risk of non-communicable diseases, to promote anti-inflammatory dietary habits and reduce chronic disease risk in this population.

## 1. Introduction

Diet plays a key role in the development and prevention of chronic diseases by influencing metabolic health and inflammatory processes [1]. Low-grade chronic inflammation is increasingly being recognized as a central mechanism in the development of many chronic diseases, such as cardiovascular disease, diabetes mellitus, chronic kidney disease, non-alcoholic fatty liver disease, autoimmune and neurodegenerative disorders [2], and certain cancers, including colorectal, hepatocellular, gastric, breast, cervical cancer, and more [3]. Beyond genetic and lifestyle factors, diet is a critical determinant of inflammatory status [4]. Specific dietary components, such as saturated fats, refined carbohydrates, and added sugars, have been shown to have pro-inflammatory effects [1], while others, such as fruits, vegetables, whole grains, nuts, legumes, spices, herbs, and plant-based protein have anti-inflammatory effects [1]. This shows that diet is not just a source of nutrients and energy but also is an important modulator of inflammation in the body.

Saudi Arabia has experienced a rapid dietary transition in recent decades, marked by urbanization and lifestyle shifts from traditional diets toward Westernized, energy-dense patterns [5]. Traditional Saudi dishes were relaying on whole grains such as whole-wheat breads, porridges, and fiber-rich foods [6]. However, modernization has driven a trend towards Westernization of eating habits, characterized by low intakes of fruits, vegetables, fish, nuts, and dairy products, alongside high intakes of meat and sugar-sweetened beverages [5]. Studies also note increasing intake of ultra-processed foods and fast foods in Saudi, replacing traditional foods [7]. These changes are important in the context of dietary inflammatory potential, as Westernized dietary patterns are typically more pro-inflammatory [4]. The ongoing shift away from traditional foods may therefore contribute to an increasing inflammatory burden in the population. This dietary shift has also corresponded with an increased burden of non-communicable diseases (NCDs), where the International Diabetes Federation estimates that roughly 23.1% of Saudi adults live with type 2 diabetes in 2024 [8]. Cardiovascular disease (CVD) is also an increasingly significant health concern. In Saudi Arabia, an estimated 236,815 individuals are living with CVD in 2024 [9], among which ischemic heart disease represented the leading cause of death in Saudi Arabia, accounting for 47.2 deaths per 100,000 in 2021 [10].

Chronic low-grade inflammation is now recognized as a central factor in the development of major NCDs, marked by elevations of hs CRP, IL 6, TNF α, IL 1β, MCP 1, and VCAM 1 [11,12,13,14], where the production of these pro-inflammatory mediators can cause tissue damage and impaired physiological function [11]. Given the established role of chronic low-grade inflammation in the development of NCDs, understanding the inflammatory potential of modern dietary patterns in Saudi Arabia is increasingly important.

Despite these trends, research on the inflammatory potential of dietary patterns in Saudi Arabia remains limited. To date, only two studies have examined this topic, both conducted exclusively among female college students, and neither explored the association between dietary inflammatory potential and sociodemographic factors [15,16], emphasizing a need for more research on the inflammatory potential of diet patterns in this region. This study aimed to assess the inflammatory potential of diet in a Saudi Arabian population and to examine its associations with sociodemographic factors.

## 2. Methods

### 2.1. Study Design and Setting

This cross-sectional study enrolled 256 residents of Saudi Arabia through convenience sampling. Recruitment was conducted using advertisements on various social media platforms, including X (formerly Twitter), Instagram, WhatsApp, and Telegram. Data collection took place between November 2024 and August 2025. Participants first completed an online forum shared via these platforms to assess eligibility and collect sociodemographic, lifestyle, and dietary data. Participants who met the eligibility criteria and consented to participate provided demographic information, including age, sex, education level, marital status, employment status, nationality, and income, as well as lifestyle factors, such as smoking and supplement use, along with weight and height. This forum also provided details about the study’s objectives, a confidentiality statement, and a consent form, which participants must review and acknowledge to proceed. Ethical approval for the study was obtained from the Research Ethics Committee of the Unit of Biomedical Ethics, King Abdulaziz University (Reference No. 193-24).

### 2.2. Study Sample

**Figure 1:**
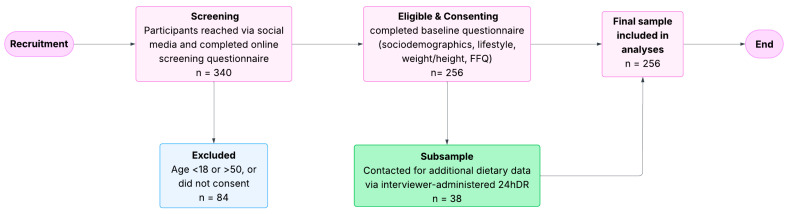
Flow chart of participant recruitment, screening, and inclusion. *n* = refers to the number of participants at each stage. FFQ = food frequency questionnaire; 24hDR = 24-hour dietary recall.

The inclusion criteria consisted of males and females aged 18–50 living in Saudi Arabia. Exclusion criteria were individuals living outside Saudi Arabia, those younger than 18 or older than 50 years, and non-Arabic speakers. No disease or medication-related exclusion criteria were applied because the study aimed to capture real-world variability in diet rather than assess clinical outcomes. Similarly, several community-based DII studies have not excluded participants based on chronic diseases or medication use [17,18]. The process of participant recruitment, eligibility screening, exclusions, and final inclusion in the study is illustrated in (Figure 1).

### 2.3. Sample Size

The required sample size was calculated using G*Power version 3.1.9.6 [19], using ANOVA: fixed effects, omnibus, one-way model, because the Kruskal–Wallis test is a nonparametric analogue of the one-way ANOVA. An α error probability of 0.05, a desired power of 0.80, and a medium effect size (Cohen’s f = 0.25) were used. The between-groups factor was set to the largest categorical predictor, region (Middle, Eastern, Western, Northern, and Southern), to ensure that the sample size would be sufficient for all demographic comparisons, the total sample size required was 200 participants. Our final sample of 256 participants therefore provided sufficient power to detect group differences.

### 2.4. Dietary Assessment

Diet history was collected using an electronic version of a food frequency questionnaire (FFQ) that has been developed and validated previously in Saudi Arabia [20]. This FFQ has also been used in previous studies in Saudi Arabia to calculate the E-DII, where it showed significant positive associations with serum hs CRP [15,16]. For each question, respondents could choose from 9 frequency options: never or less than once a month, 1–3 times per month, once a week, 2–4 times per week, 5–6 times per week, once a day, 2–3 times per day, 4–5 times per day, or 6+ times per day. This FFQ includes 133 food items to measure habitual food consumption in the past 6 months. Additional data on specific food items (green or black tea, garlic, ginger, pepper, thyme or oregano, rosemary, turmeric, and saffron) was added as it is required for the assessment of the DII. The FFQ also includes a section with open-ended questions about supplement use, including details on the types of supplements and the amount consumed. Standardized portion sizes tailored to each food item in the FFQ were used to estimate individual food intakes. The overall composition of participants’ diets was calculated using Nutritics software (Nutritics, 2019) [21], which has been used previously in Saudi Arabia, and includes a national food database for universal Arabic foods [22]. To estimate the intake of flavones, flavanones, flavon-3-ols, flavanols, and anthocyanidins, we used the U.S. Department of Agriculture database for the flavonoid content of selected foods [23]. To account for measurement error, 15% of the participants also completed a 24-hour dietary recall (24hDR), which was used as a reference method to calibrate FFQ-reported intake using linear regression [24]. The 24hDR was administered by a trained dietitian using an interviewer-administered approach. Each recall represented a single day, and participants were asked to confirm whether the reported day was typical of their usual intake and not influenced by occasions or unusual circumstances. For each nutrient, a linear regression was fitted with 24hDR intake as the dependent variable and FFQ intake as the independent variable. The resulting slope (*β*) was then used to adjust FFQ values according to the equation:

$$\mathrm{Calibrated}\;\mathrm{FFQ}\;\mathrm{intake}=\mathrm{mean}\;\mathrm{FFQ}+\beta \times \left(\mathrm{individual}\;\mathrm{FFQ}\;-\;\mathrm{mean}\;\mathrm{FFQ}\right)$$

These calibrated nutrient values were then used to calculate the E-DII, improving the accuracy of dietary assessment. This approach allowed for the assessment of habitual intake while minimizing participant burden. The calibrated FFQ values were used in the analysis of nutrient intake.

### 2.5. Dietary Inflammatory Index (DII)

The DII is a widely used tool in the literature to assess the inflammatory potential of a diet and has been used in numerous studies to investigate the association between dietary patterns and various chronic diseases [25]. It is designed to measure the overall inflammatory potential of a diet by assigning scores to different dietary components based on scientific evidence. The DII considers a wide range of nutrients and bioactive compounds, including macronutrients like carbohydrates, proteins, and fats, as well as vitamins, minerals, and compounds such as flavonoids, which have been shown to influence inflammation. Each component is included due to its documented pro- or anti-inflammatory effects. In this study, the DII was calculated using all available food parameters from the original DII model, except for alcohol, eugenol, and isoflavones, which were not present in our dataset. Each nutrient’s intake is first energy-adjusted using the density method (expressed per 1000 kcal of total daily energy intake) to account for differences in total energy intake. The energy-adjusted intakes were then used to calculate the energy-adjusted Dietary Inflammatory Index (E-DII) following the standard scoring algorithm. For each nutrient, intake values were converted into Z-scores and centered percentiles. Each percentile is then multiplied by a coefficient representing that nutrient’s inflammatory effect. This produces a score for each dietary component, reflecting its contribution to the diet’s overall inflammatory potential. The final E-DII score is obtained by summing all individual component scores, providing a comprehensive measure of the diet’s inflammatory potential. A positive E-DII score indicates a pro-inflammatory dietary pattern, while a negative E-DII score suggests an anti-inflammatory dietary pattern. The DII has been shown to be a useful tool for reflecting inflammatory biomarkers in different populations [25,26,27,28]. Additionally, the DII has shown a positive association with serum hs CRP levels in earlier studies conducted in Saudi Arabia [15,16].

### 2.6. Statistical Analysis

All statistical analyses were performed using IBM SPSS software (IBM SPSS Statistics, Version 30.0.0.0, 2020) [29]. Participants’ characteristics are presented using descriptive statistics. Continuous variables are expressed as means and standard deviations (SD) for descriptive purposes, while binary and categorical variables are expressed as counts and percentages. The E-DII was assessed for normality using the Shapiro–Wilk test and was found to be skewed. Therefore, for statistical comparisons, E-DII is presented as median and interquartile range (IQR).

The Mann–Whitney U test was used to compare E-DII across binary categorical variables (sex, smoking status, nationality, and supplement use), whereas the Kruskal–Wallis test was used for categorical variables with more than two groups (age group, income level, education level, employment status, marital status, BMI categories, and region of residence). A *p* < 0.05 was considered statistically significant.

## 3. Results

### 3.1. Sample Characteristics

Table 1 provides an overview of the sociodemographic characteristics of the study participants. The study included 256 participants, with females making up 71% of the sample. The age distribution was relatively balanced, with the 40–50 years age group having the smallest proportion at 24%. In terms of BMI, 41% of participants had a normal weight, while 31% were classified as overweight, and 22% were classified as obese. Regarding social and economic characteristics, 52% of participants were single. Most had a bachelor’s degree (67%) and were employed (59%). Income levels were relatively balanced, with the largest proportion (38%) falling within the middle range of 4000–10,000 SAR. Lifestyle factors showed that 89% of participants were non-smokers. Saudis made up 82% of the sample, and most participants resided in the Western region (72%). The mean E-DII was 4.8 ± 1.3, indicating an overall pro-inflammatory dietary pattern in the sample.

Table 2 presents the mean and SD of the individual components contributing to the E-DII per 1000 kcal, demonstrating the dietary intake profile of the study participants. Trends in E-DII components across tertiles were variable. Some pro-inflammatory components, such as total fat and saturated fatty acids, increased across tertiles, whereas others, such as energy, carbohydrates, protein, trans fatty acids, PUFA, cholesterol, iron, and vitamin B12 intake showed a decrease in the higher tertile. Similarly, some anti-inflammatory components, such as fiber, omega 3 fatty acids, magnesium, zinc, selenium, beta-carotene, vitamin A, D, E, C, B6, thiamin, riboflavin, niacin, folic acid, flavan-3-ols, flavanones, flavones, flavanols, onions, garlic, ginger, saffron, tea, and rosemary intake, decreased across tertiles, while others, such as MUFA, omega 6 fatty acids, caffeine, anthocyanidins, turmeric, and pepper intake increased across tertiles.

### 3.2. Relationship Between Sociodemographic Data and E-DII

The associations between sociodemographic and lifestyle characteristics and the E-DII are presented in Table 3. There was a significant difference in E-DII scores by sex (U = 4734, *p* < 0.001), with males having a higher median E-DII score (5.7, IQR 1.2) compared to females (4.9, IQR 2.0). For education level, the E-DII scores tended to be lower for participants with postgraduate degrees (4.9, IQR 2.1) and higher for participants with only high school degrees (5.7, IQR 1.3); however, this difference was not statistically significant (*p* = 0.06). No significant difference in E-DII was found across age, income level, education level, employment status, marital status, smoking status, BMI category, region of residence, or nationality.

## 4. Discussion

### 4.1. The Main Findings

This study examined the association between sociodemographic and lifestyle factors and dietary inflammatory potential, measured by the E-DII, among adults in Saudi Arabia. The study revealed that there was a significant difference in E-DII scores observed across sex, with males exhibiting higher E-DII scores than females, indicating a more pro-inflammatory diet. The overall dietary inflammatory potential of the participants was pro-inflammatory, with a mean E-DII of 4.8 ± 1.3. No significant difference in E-DII was found across age, income level, education level, employment status, marital status, smoking status, BMI category, region of residence, or nationality These findings indicate that sex is an important sociodemographic factor influencing pro-inflammatory diet in this sample.

### 4.2. Comparison with Other Studies

This study found a significant difference in E-DII scores by sex, with males having higher E-DII scores, indicating a more pro-inflammatory diet. This finding aligns with several previous studies conducted in Asia and Africa, where men were more likely than women to follow a more pro-inflammatory diet [30,31,32]. Similarly, in Iran, men had significantly higher DII scores compared to women [33]. These differences are often linked to lifestyle and behavioral factors, such as higher levels of physical activity, occupational demands, and variations in nutrient intake [34,35]. For example, higher physical activity and longer working hours, often observed in men, could lead them to neglect healthier dietary choices [34,36]. Moreover, males have been reported to have higher intake of pro-inflammatory foods such as red and processed meat, fried foods, and other energy-dense foods, alongside lower consumption of fruits, vegetables, and whole grains [37]. In addition, males tend to eat outside the home more frequently, have a faster eating pace, and lower adherence to health-conscious dietary practices [38], further contributing to the higher inflammatory potential of their diets. Studies in the United States have found controversial results. Some studies reported that women tend to have more pro-inflammatory diets compared to men, often linked to hormonal fluctuations such as premenstrual syndrome (PMS), which can cause an increased appetite and cravings for energy-dense, pro-inflammatory foods [39,40,41,42]. In contrast, other studies found men to be more exposed to pro-inflammatory diet patterns due to higher consumption of energy-dense, nutrient-poor foods and snacks [43].

The E-DII scores tended to be lower for participants with postgraduate degrees and higher for participants with only high school degrees; however, this difference was not statistically significant (*p* = 0.06). This pattern is similar to several studies reporting that individuals with higher education tend to have significantly lower E-DII scores, reflecting a less pro-inflammatory diet [44,45,46]. Socioeconomic status, including education, is linked to better diet quality, where individuals of higher socioeconomic status tend to have better diet quality, characterized by consumption of more fruits, vegetables, whole grains, fish, fiber, and less red, processed meats, sugary drinks, saturated fats, and sodium [47,48]. One factor contributing to lower E-DII scores among individuals with higher education is that education enables better comprehension of health messages, more educated individuals are better at understanding and applying health-related information, including nutrition guidelines [49], which can lead to more informed dietary choices. Nutrition literacy is also a key factor influencing dietary choices. Higher education is often associated with greater health and better nutrition literacy [48,50,51], allowing individuals to interpret dietary information, understand food labels, and apply nutritional knowledge in daily meal planning [48]. Evidence also suggests that nutrition literacy mediates the relationship between education and diet quality, with more educated individuals being more likely to translate their knowledge into healthier eating patterns [52]. Some dietary behaviors that are often reported among undergraduate students can affect dietary patterns. For example, studies done in Saudi Arabia and Jordan have shown a high prevalence of meal skipping, and low consumption of fruits and vegetables among students, with many reporting intakes of these foods fewer than three times per week [53,54,55]. Similarly, studies among Saudi female college students, of whom 89% were bachelor’s students, reported high E-DII scores, reflecting a pro-inflammatory diet pattern [15,16]. Globally, similar trends have been observed, where studies from the United States and Europe reported that college students consume less than recommended servings of fruits and vegetables, and heavily rely on fast foods [56,57,58]. In Saudi Arabia, high availability of fast food, high cost of healthy food, limited time, and lack of motivation were the main barriers reported for following healthy diet patterns among university students [55]. The lack of significant association between education level and E-DII scores in our study could be due to the small proportion of participants with an only high school degree (8%).

This study found an overall pro-inflammatory dietary pattern among participants. Previous research in Saudi Arabia similarly reported pro-inflammatory diets, with mean E-DII scores of 3.90 ± 1.08 [16,17]. This pro-inflammatory trend was also observed in neighboring countries, including Bahrain, where the mean E-DII score was 1.79 ± 1.52 [59], the United Arab Emirates, with a mean E-DII score of 2.98 ± 1.17 [60], and Jordan, reporting a mean DII score of 1.5 ± 1.0 [61]. Differences in reported E-DII scores could be due to methodological differences. Specifically, E-DII can differ based on the number of parameters used for score calculation, for example, when all 45 parameters are used for E-DII calculation, the expected E-DII score can range between −8.87 to +7.98, while using 25–30 parameters can produce a score range of 5.5 to +5.5 [25]. Many of these studies did not include key components in calculating the E-DII, such as Monounsaturated Fatty Acids (MUFA), Polyunsaturated Fatty Acids (PUFA), trans fats, omega 6 fatty acids, B-vitamins, Magnesium, Zinc, flavonoids, and spices. In contrast, this study included 42 of the 45 DII components. Another factor could be the study population, previous studies focused on specific groups, such as female college students or patients with schizophrenia, rather than the general adult population. In this study, we provided a more comprehensive representation of the pro-inflammatory dietary pattern among the general adult population in Saudi Arabia. These reasons could explain the differences in E-DII scores reported previously.

The dietary inflammatory potential of diet, as measured using the DII, differs between different regions, reflecting eating habits. For example, diets rich in fruits, vegetables, and unsaturated fats, such as traditional Mediterranean or East Asian coastal diets, tend to produce low or anti-inflammatory scores [62], while western-style diets high in red meat and processed foods usually produce higher or pro-inflammatory scores [62]. Similarly, a global review reports that older adults in parts of South America, East Asia, and the Arab countries had more pro-inflammatory diets, whereas traditional diets in other cultures can be more anti-inflammatory [63]. These findings emphasize that dietary patterns in different regions lead to population differences in average DII scores. Numerous studies demonstrate that sociodemographic factors significantly influence diet quality [64], which is, consequently, the inflammatory potential of a diet. In the U.S. NHANES data, more than half of adults had pro-inflammatory diets, and the highest scores were observed in men, younger adults, and non-Hispanic Black individuals [65]. Conversely, women, older adults, Hispanic individuals, and those with higher education or income had the most anti-inflammatory diets [65]. Globally, men were observed to have higher pro-inflammatory diets than women [63]. Moreover, fewer years of education or lower income are linked to more pro-inflammatory diets [63,65]. Lifestyle behaviors also are associated with the inflammatory potential of diet, for example, smokers and heavy drinkers appear to have more pro-inflammatory patterns than non-users [63]. Findings from a study on female Saudi college students indicated that their average DII score was pro-inflammatory [15,16]. This pattern is also observed in Bahrain [59], Jordan [61], and in the United Arab Emirates [60].

The DII has been shown to be a useful tool for reflecting inflammatory biomarkers in different populations. For example, in the Women’s Health Initiative Observational Study, a subsample of 2567 postmenopausal women showed that higher DII scores were significantly associated with IL 6, TNF α Receptor 2, and hs CRP [66]. Similarly, in the Seasonal Variation of Blood Cholesterol Study, higher DII scores were associated with hs CRP concentrations >3 mg/L [28]. In a large Japanese cohort, the E-DII was positively associated with hs CRP concentration in men, although the association was limited in women [26]. Additionally, analyses from the Moli-sani population-based study of over 20,000 adults showed that higher DII scores were directly associated with a composite score of low-grade inflammation, including CRP, platelet, and leukocyte counts, and granulocyte-to-lymphocyte ratio [27]. Collectively, these findings support the utility of the DII as a valid tool for capturing the inflammatory potential of diet and align with earlier studies conducted in Saudi Arabia that reported a positive association between DII scores and serum hs CRP levels [15,16].

Overall, these findings highlight the pro-inflammatory dietary patterns among adults living in Saudi Arabia and indicate that demographic factors such as sex, and potentially education, may influence dietary inflammatory potential.

### 4.3. Limitations

Several limitations of this study should be addressed. The study sample was mostly female, Saudi, and from the western region, which may limit the generalizability of the findings to broader populations. Additionally, the cross-sectional design provides a snapshot of dietary patterns across different sociodemographic and lifestyle factors but cannot account for changes over time. Longitudinal studies are needed to understand more about how sociodemographic and lifestyle factors influence dietary patterns over time. Furthermore, dietary intake was assessed using an FFQ and 24hDR, which could be affected by self-report bias. Moreover, under- or over-reporting of pro-inflammatory and anti-inflammatory items such as herbs and spices could have influenced the E-DII scores. Additionally, the convenience sampling method may cause selection bias, as participation was voluntary and limited to individuals who chose to respond to the online survey, the study sample may differ in unmeasured ways from the broader population, limiting generalizability. Another limitation of this study is the absence of inflammatory biomarkers such as hs CRP and IL 6, which prevents direct comparison with physiological inflammation. Instead, the E-DII was used as a proxy indicator of inflammatory potential rather than a measure of actual inflammatory status. Finally, some subgroups, such as certain regions or BMI categories, had small sample sizes, which may reduce statistical power to detect differences.

### 4.4. Implications

The findings reported in this study have several implications for public health and policy. Specific groups, such as males who are at higher risk of non-communicable diseases (for example, those with obesity, hypertension, diabetes, or a family history of cardiovascular disease), may benefit from targeted dietary interventions. Dietary recommendations should focus on increasing the consumption of fruits, vegetables, fish, whole grains, legumes, and nuts, as well as incorporating herbs and spices, such as ginger, thyme, garlic, saffron, turmeric, and tea, while reducing the intake of red and processed meats, fast food, fried foods, and sugar-sweetened beverages. Furthermore, promoting tailored nutrition education programs, public health initiatives, and supportive policies that improve access to healthy foods for these higher-risk groups may help reduce diet-related inflammation, lower the risk of associated health conditions, and improve general health. Future research should evaluate the effectiveness of such interventions and assess the long-term effects on the risk of associated health conditions.

## 5. Conclusions

This study found that males had significantly higher pro-inflammatory dietary patterns, while other sociodemographic and lifestyle factors were not significantly associated with pro-inflammatory dietary patterns. These findings suggest that dietary interventions could benefit from being tailored to sex. Promoting anti-inflammatory foods and appropriate dietary changes could help improve overall diet quality and support long-term health outcomes. Future studies with larger populations and longitudinal designs are needed to confirm these results and guide targeted nutrition strategies.

## Figures and Tables

**Table 1:** Descriptive Characteristics of Participants.

Characteristic	*N*	%
**Sex**		
Males	76	29
Females	180	71
**Age Group**		
18–29 y	116	45
30–39 y	79	31
40–50 y	61	24
**Education Level**		
High school	20	8
Bachelor’s	171	67
Postgraduate	65	25
**Employment status**		
Unemployed	70	27
Employed	149	59
Student	37	14
**Income Level**		
<4000 SAR	95	37
4001–10,000 SAR	98	38
>10,001 SAR	63	25
**Region of Residence**		
Middle	32	13
Western	186	72
Eastern	9	4
Southern	18	7
Northern	11	4
**Marital status**		
Single	133	52
Married	110	43
Separated	13	5
**Nationality**		
Saudi	210	82
Non-Saudi	46	18
**Smoking Status**		
Yes	29	11
No	227	89
**BMI Category**		
Underweight	15	6
Normal	106	41
Overweight	80	31
Obese	55	22

Note. SAR, Saudi Riyal; BMI, body mass index.

**Table 2:** Distribution of Dietary Components Intake Across E-DII Tertiles.

Dietary Component	T1	T2	T3
E-DII	2.5 ± 1.1	4.4 ± 0.3	5.0 ± 0.1
Energy (kcal)	1728.0 ± 216.1	1641.1 ± 67.1	1612.9 ± 29.5
Carbohydrates (g)	196.8 ± 41.7	179.6 ± 12.8	174.2 ± 5.7
Protein (g)	83.6 ± 17.4	76.7 ± 5.4	74.3 ± 2.3
Total Fat (g)	62.1 ± 5.8	64.3 ± 1.9	65.1 ± 0.9
Fiber (g)	14.6 ± 1.8	13.9 ± 0.6	13.6 ± 0.2
Saturated Fatty Acids (g)	17.5 ± 4.6	19.4 ± 1.7	20.1 ± 0.8
MUFA (g)	18.5 ± 2.9	19.6 ± 1.0	20.0 ± 0.5
PUFA (g)	15.1 ± 3.8	13.8 ± 1.3	13.4 ± 0.8
Omega 3 Fatty Acids (g)	1.5 ± 0.6	1.3 ± 0.2	1.2 ± 0.2
Omega 6 Fatty Acids (g)	6.6 ± 0.4	6.5 ± 0.2	6.4 ± 0.1
Trans Fat (g)	1.0 ± 0.9	0.7 ± 0.3	0.6 ± 0.2
Cholesterol (g)	442.0 ± 323.3	315.8 ± 98.6	276.6 ± 44.9
Magnesium (mg)	267.4 ± 88.7	211.3 ± 58.9	198.1 ± 1.3
Iron (mg)	26.9 ± 12.2	13.2 ± 5.2	11.8 ± 5.0
Zinc (mg)	13.7 ± 5.9	6.9 ± 1.7	6.4 ± 0.2
Selenium (mg)	141.9 ± 43.8	101.1 ± 12.2	96.0 ± 5.5
Vitamin A (RE)	427.9 ± 405.4	293.3 ± 178.8	231.4 ± 41.2
b-Carotene (mg)	417.0 ± 658.8	189.3 ± 345.0	2.1 ± 8.8
Vitamin D (μg)	15.2 ± 9.7	7.9 ± 10.7	1.7 ± 0.2
Vitamin E (mg)	11.7 ± 7.8	6.0 ± 7.2	5.4 ± 0.2
Thiamin (mg)	6.9 ± 14.1	1.8 ± 5.3	1.1 ± 0.1
Riboflavin (mg)	6.7 ± 13.2	1.4 ± 0.4	1.2 ± 0.2
Niacin (mg)	48.3 ± 16.5	31.6 ± 4.4	29.6 ± 1.8
Vitamin B6 (mg)	7.7 ± 13.2	3.6 ± 7.5	2.4 ± 0.2
Folic Acid (mg)	546.4 ± 221.1	251.2 ± 62.4	223.9 ± 18.5
Vitamin B12 (mg)	10.4 ± 13.2	3.9 ± 1.6	3.9 ± 0.9
Vitamin C (mg)	61.0 ± 29.2	29.5 ± 1.0	29.0 ± 0.5
Caffeine (mg)	53.8 ± 7.5	56.3 ± 3.0	56.0 ± 4.7
Anthocyanidins (mg)	2.6 ± 1.4	3.3 ± 0.8	3.6 ± 0.3
Flavan-3-ol (mg)	64.5 ± 80.2	28.3 ± 26.6	18.8 ± 11.5
Flavanones (mg)	3.6 ± 2.4	4.3 ± 2.1	5.3 ± 1.0
Flavones (mg)	3.7 ± 2.0	3.0 ± 1.0	2.4 ± 0.0
Flavanols (mg)	11.8 ± 3.9	10.1 ± 1.4	9.3 ± 0.6
Onion (g)	43.9 ± 36.2	31.9 ± 29.8	26.2 ± 30.9
Garlic (g)	6.1 ± 7.9	3.5 ± 4.1	3.6 ± 5.7
Ginger (g)	1.5 ± 2.3	0.6 ± 0.9	0.4 ± 0.8
Saffron (g)	0.6 ± 1.1	0.2 ± 0.3	0.1 ± 0.2
Turmeric (mg)	41.1 ± 33.8	57.6 ± 28.9	65.2 ± 24.9
Green/Black Tea (g)	2.5 ± 3.2	2.2 ± 2.6	1.4 ± 1.6
Pepper (g)	3.0 ± 3.6	1.5 ± 2.0	1.8 ± 2.1
Thyme/Oregano (mg)	1633.5 ± 2302.3	596.9 ± 885.1	454.3 ± 1002.0
Rosemary (mg)	550.5 ± 977.7	184.4 ± 342.5	63.6 ± 167.7

Note. All dietary component intakes are standardized per 1000 kcal of total energy intake. E-DII was calculated using 42 out of 45 parameters. Alcohol was excluded due to the religious background of the sample, while eugenol and isoflavones were omitted due to data unavailability. Abbreviations: E-DII, Energy-adjusted dietary inflammatory index; MUFA, Monounsaturated Fatty Acids; PUFA, Polyunsaturated Fatty Acids.

**Table 3:** Distribution of E-DII Scores by Sociodemographic and Lifestyle Characteristics.

Characteristic	*N*	Median (IQR) E-DII	Test Statistic	*p*-Value
**Sex**			U = 4734	<0.001 **
Male	76	5.7 (1.2)		
Female	180	4.9 (2.0)		
**Age group**			H = 0.09	0.95
18–29 y	116	5.1 (1.7)		
30–39 y	79	5.0 (1.5)		
40–50 y	61	5.1 (1.8)		
**Education Level**			H = 5.67	0.06
High school	20	5.7 (1.3)		
Bachelor’s	171	5.1 (1.6)		
Postgraduate	65	4.9 (2.1)		
**Employment status**			H = 0.23	0.89
Unemployed	70	5.0 (1.6)		
Employed	149	5.1 (1.6)		
Student	37	5.1 (1.7)		
**Income Level**			H = 2.26	0.32
<4000 SAR	95	4.8 (1.8)		
4001–10,000 SAR	98	5.2 (1.5)		
>10,001 SAR	63	5.1 (1.6)		
**Region of Residence**			H = 6.04	0.20
Middle	32	4.8 (1.3)		
Western	186	5.1 (1.6)		
Eastern	9	5.5 (2.9)		
Southern	18	5.4 (1.3)		
Northern	11	4.4 (1.5)		
**Marital Status**			H = 3.92	0.14
Single	133	5.1 (1.6)		
Married	110	5.1 (1.7)		
Separated	13	5.0 (2.0)		
**Nationality**			U = 4495	0.46
Saudi	210	5.1 (1.6)		
Non-Saudi	46	4.8 (1.6)		
**Smoking Status**			U = 3053	0.53
Yes	29	5.1 (1.7)		
No	227	5.0 (1.6)		
**BMI Category**			H = 1.95	0.58
Underweight	15	5.7 (1.5)		
Normal	106	5.0 (1.5)		
Overweight	80	4.9 (1.9)		
Obese	55	5.2 (1.3)		

Note. Comparisons across two-group variables were performed using the Mann–Whitney U test, and comparisons across variables with more than two groups were performed using the Kruskal–Wallis test. Abbreviations: E-DII, energy-adjusted dietary inflammatory index; SAR, Saudi Riyal; BMI, body mass index; IQR, interquartile range. ** *p* value < 0.05 indicates a statistically significant association.

## Data Availability

The original contributions presented in this study are included in the article. Further inquiries can be directed to the corresponding author.

## Abbreviations

The following abbreviations are used in this manuscript:

24hDR	24-hour dietary recall
ANOVA	Analysis of variance
BMI	Body mass index
CVD	Cardiovascular disease
DII	Dietary inflammatory index
E-DII	Energy-adjusted dietary inflammatory index
FFQ	Food Frequency Questionnaire
hs CRP	High-sensitivity C-reactive protein
IL	Interleukin
IQR	Interquartile
MUFA	Monounsaturated fatty acids
NCDs	Noncommunicable diseases
PMS	Premenstrual syndrome
PUFA	Polyunsaturated fatty acids
SD	Standard deviation
TNF	Tumor necrosis factor
